# A display of pH-sensitive fusogenic GALA peptide facilitates endosomal escape from a Bio-nanocapsule via an endocytic uptake pathway

**DOI:** 10.1186/1477-3155-12-11

**Published:** 2014-04-01

**Authors:** Yuya Nishimura, Koichi Takeda, Ryosuke Ezawa, Jun Ishii, Chiaki Ogino, Akihiko Kondo

**Affiliations:** 1 Organization of Advanced Science and Technology, Kobe University, 1-1 Rokkodaicho, Nada-ku, Kobe 657-8501, Japan; 2 Department of Chemical Science and Engineering, Graduate School of Engineering, Kobe University, 1-1 Rokkodaicho, Nada-ku, Kobe 657-8501, Japan

**Keywords:** Bio-nanocapsule, pH-sensitive peptide, GALA, Endosomal escape, HER2, Drug delivery

## Abstract

**Background:**

An affibody-displaying bio-nanocapsule (Z_HER2_-BNC) with a hepatocyte specificity derived from hepatitis B virus (HBV) was converted into an affibody, Z_HER2_, that recognizes HER2 receptors. This affibody was previously reported to be the result of the endocytosis-dependent specific uptake of proteins and siRNA into target cancer cells. To assist the endosomal escape of inclusions, a helper lipid with pH-sensitive fusogenic ability (1,2-dioleoyl-sn-glycero-3-phos phoethanolamine; DOPE) was conjugated with a Z_HER2_-BNC.

**Findings:**

In this study, we displayed a pH-sensitive fusogenic GALA peptide on the surface of a particle in order to confer the ability of endosomal escape to a Z_HER2_-BNC. A GALA-displaying Z_HER2_-BNC purified from yeast uneventfully formed a particle structure. Furthermore, endosomal escape of the particle was facilitated after endocytic uptake and release of the inclusions to the cytoplasm without the cell toxicity.

**Conclusion:**

The genetic fusion of a GALA peptide to the virus-like particle confers the ability of endosomal escape.

## Background

Target-cell specificity and effective delivery are essential attributes for a successful drug delivery system (DDS). However, lysosomal degradations of the drugs after endocytic entry into the cells presents barriers that are often insurmountable. To increase the curative effects, it is necessary to release the drugs trapped in endosomes by clathrin- or caveola-mediated endocytosis to the cytoplasm [1,2].

To solve this problem, membrane fusion peptides existing on virus envelopes have attracted attention. For example, influenza virus has an amphiphilic anionic peptide that is referred to as HA2, and has the ability to escape from endosomes. While HA2 has a nonhelical structure in neutral pH, it changes to a helical secondary structure under protonated conditions. This helix structure promotes fusion with an endosomal membrane, which results in the release of inclusions into the cytoplasm [3].

Comparable synthetic peptides have also been developed to mimic the fusogenic functions of virus-derived peptides [2]. The most famous synthetic peptide, the GALA, has pH-sensitive and amphiphilic features. The GALA consists of 30 amino acid residues (WEA ALA EAL AEA LAE HLA EAL AEA LEA LAA), and has a repeating sequence of Glu, Ala, Leu, and Ala. A random coil structure is formed by the repulsive force caused by the negative charge of Glu residues at pH 7, whereas an amphipathic α helix structure is formed by the partially protonated Glu residues with membrane fusion activity appearing at pH 5. Thus, GALA could induce the assistive process to release the drugs from endosomes in common with other virus-derived fusogenic peptides [4,5].

A bio-nanocapsule (BNC) that consists of a hepatitis B surface antigen (HBsAg) and a lipid bilayer is a hollow virus-like particle [6] that has been studied as a carrier for DDS [7]. Since a wild type BNC recognizes hepatic cells specifically, it has been used to deliver drugs to hepatocarcinomas [7]. In the past, a wild type BNC has been engineered to recognize other types of cells by replacing the hepatocyte recognition site in the pre-S region of an L protein with other targeting molecules [8-10]. For example, a Z_HER2_ affibody displaying BNC (Z_HER2_-BNC) recognizes specific HER2-expressing cells such as breast cancer and ovarian cancer cells [11]. The Z_HER2_ is one of a type of affibodies that are the mutant proteins derived from the Z domain of Staphylococcal protein A and function as affinity ligands [12,13]. Thus far, the Z_HER2_-BNC has succeeded in specific deliveries and function expressions of proteins or siRNA to the target cells by conjugating to a mixture of a specialized, cationic liposome (LP) with the ability for endosomal escape (1,2-dioleoyl-sn-glycero-3-phos phoethanolamine; DOPE) and an anionic LP [14,15].

In the present study, we intended to bestow the ability for endosomal escape into a specificity-altered BNC (Z_HER2_-BNC) to avoid the use of a positively charged LP containing DOPE. To achieve this, a GALA peptide was used as the fusogenic peptide. To display a GALA peptide on the surface of Z_HER2_-BNC, it was genetically fused to a Z_HER2_-L protein.

## Findings

**Figure 1:**
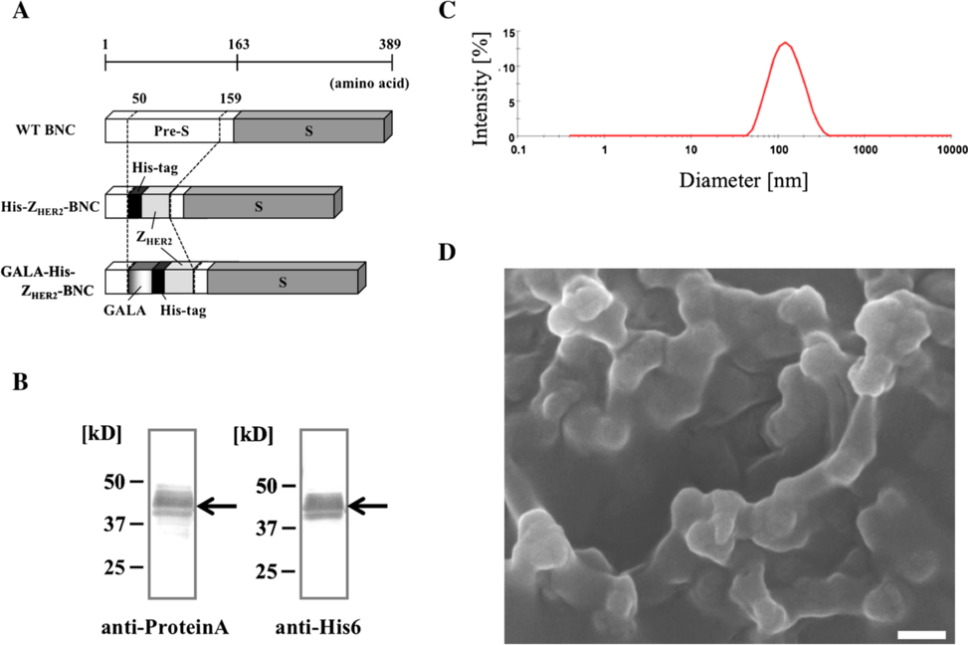
**Construction and characterization of GALA-His-Z**_**HER2**_**-BNC. (A)** Schematic representations of constructed His-Z_HER2_-BNC and GALA-His-Z_HER2_-BNC. The plasmid for expression of the GALA-fused His6-Z_HER2_-BNC (GALA-His-Z_HER2_-BNC) was constructed by replacing the His-Z_HER2_ fragment with a GALA-His-Z_HER2_ fragment. Briefly, a DNA fragment encoding the GALA was prepared by annealing A1 and A2 primer pairs (A1; 5'- GGG GGA TCC TGG GAA GCT GCT TTG GCT GAA GCT TTG GCT GAA GCT TTG GCT GAA CAC TTG GCT GAA GCT TTG GCT GAA GCT TTG GAA GCT TTG GCT GCT -3' and A2; 5'- GTG GTG GTG AGC AGC CAA AGC TTC CAA AGC TTC AGC CAA AGC TTC AGC CAA GTG TTC AGC CAA AGC TTC AGC CAA AGC TTC AGC CAA AGC AGC TTC CCA -3'). A DNA fragment encoding the His-Z_HER2_ was amplified by PCR with B1 and B2 primer pairs (B1; 5'- TTT GGC TGC TCA CCA CCA CCA CCA CCA CGC GCA ACA CG AT -3' and B2; 5'- GGG GCG GCC GCC TTT CGG CGC CTG AGC ATC AT -3') from pGLDsLd50-His-Z_HER2_[16]. A DNA fragment encoding the entire GALA-His-Z_HER2_ was amplified by an overlap PCR of the amplified fragments with A1 and B2 primer pairs, and was digested with *BamH*I/*Not*I and ligated into the same sites of pGLDsLd50-His-Z_HER2_. The resultant plasmid was designated as a pGLDsLd50-GALA-His-Z_HER2_. **(B)** Western blotting analyses of GALA-His-Z_HER2_-BNC. Sample was subjected to SDS-PAGE followed by immune blotting using anti-His6 antibody (for His6 tag; left image) and anti-protein A antibody (for Z_HER2_ affibody; right image). **(C)** Size distribution using DLS analysis. The average size of the GALA-His-Z_HER2_-BNC was 105 nm. **(D)** Scanning electron microscope image of GALA-His-Z_HER2_-BNC. GALA-His-Z_HER2_-BNC freeze-dried with sucrose was analyzed using a JSM-7500 F (JEOL, Munchen, Germany), following the manufacturer's procedure. Scale bar: 100 nm.

To accomplish endosomal escape, the fusion was designed to orient the GALA peptide in the direction of the outer surface of the Z_HER2_-BNC (including a His6 tag for purification). Thus, the GALA was fused to the N-terminus of a His6-Z_HER2_ fusion protein (GALA-His-Z_HER2_-BNC) (Figure 1A).

A yeast *Saccharomyces cerevisiae* AH22R^-^ strain was transformed with the constructed plasmid using the spheroplast method, and was cultured and disrupted with glass beads [6]. The GALA-His-Z_HER2_-BNC in the crude extract was purified via His6 affinity chromatography [16]. Then, to establish whether the obtained band was definitely GALA-His-Z_HER2_ fusion proteins, we performed western blotting using anti-His6 and anti-protein A antibodies (Figure 1B). When the coincident bands were detected in both cases, this confirmed that the BNC contained purified GALA-His-Z_HER2_ fusion protein. Furthermore, to examine whether the GALA-His-Z_HER2_-BNC formed a particle structure, we measured the diameter by dynamic light scattering (DLS) using a Zetasizer Nano particle size analyzer (Malvern Instruments, Worcestershire, UK) (Figure 1C). The diameter of the GALA-His-Z_HER2_-BNC was about 100 nm and was similar to that of a His-Z_HER2_-BNC [16]. Furthermore, the particle structure of the GALA-His-Z_HER2_-BNC was observed using scanning electron microscope (SEM) (Figure 1D). In sucrose that prevents aggregation of particles, spherical particles in a size of about 100 nm were confirmed. These results indicated that the insertion of the GALA peptide into the Z_HER2_-BNC had no influence on particle formation.

**Figure 2:**
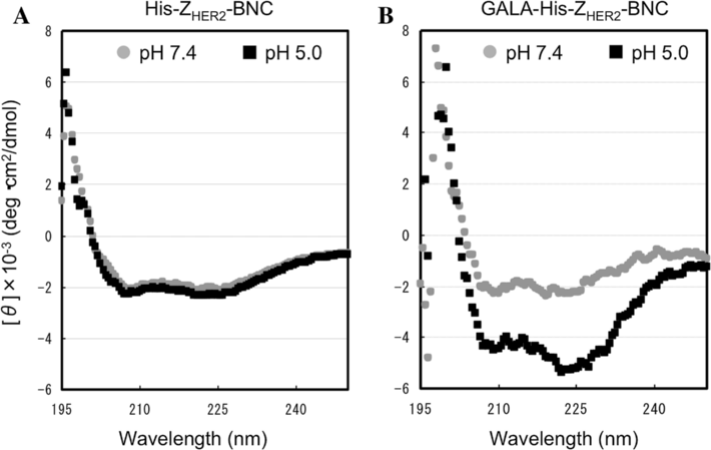
**Circular Dichroism spectra analysis of (A) His-Z**_**HER2**_**-BNC and (B) GALA-His-Z**_**HER2**_**-BNC.** Circular dichroism (CD) measurements were carried out with a J-725 K (JASCO, Japan). Spectra were obtained using 0.5 nm bandwidth, a scan rate of 20 nm/min and a response time of 4 sec. The quartz cuvette pathlength was 2 mm. The CD measurements were made using protein concentrations of 0.1 mg/ml and performed at 20°C. The gray circle and the black square show the CD measurements of BNCs after incubation of 1 h in PBS at pH 7.4 and 5.0, respectively.

To confirm if the GALA peptide displayed on the surface of Z_HER2_-BNC in a functional structure, circular dichroism (CD) spectra of His-Z_HER2_-BNC and GALA-His-Z_HER2_-BNC were measured at pH 7.4 and 5.0 (Figure 2). In the case of His-Z_HER2_-BNC, negative maxima at 208 nm and 222 nm of α-helix were same between pH 7.4 and pH 5.0 (Figure 2A). The GALA-His-Z_HER2_-BNC at pH 7.4 showed negative maximum at 195 nm that is characteristic to random coil structure. However, the GALA-His-Z_HER2_-BNC at pH 5.0 displayed the relatively stronger negative maxima 208 nm and 222 nm (Figure 2B). These results indicated that the GALA peptide on the Z_HER2_-BNC changed in the structure from random coil to α-helix responding to the pH decrease, which is an important feature that the GALA shows the pH-sensitive activity for endosomal escape.

**Figure 3:**
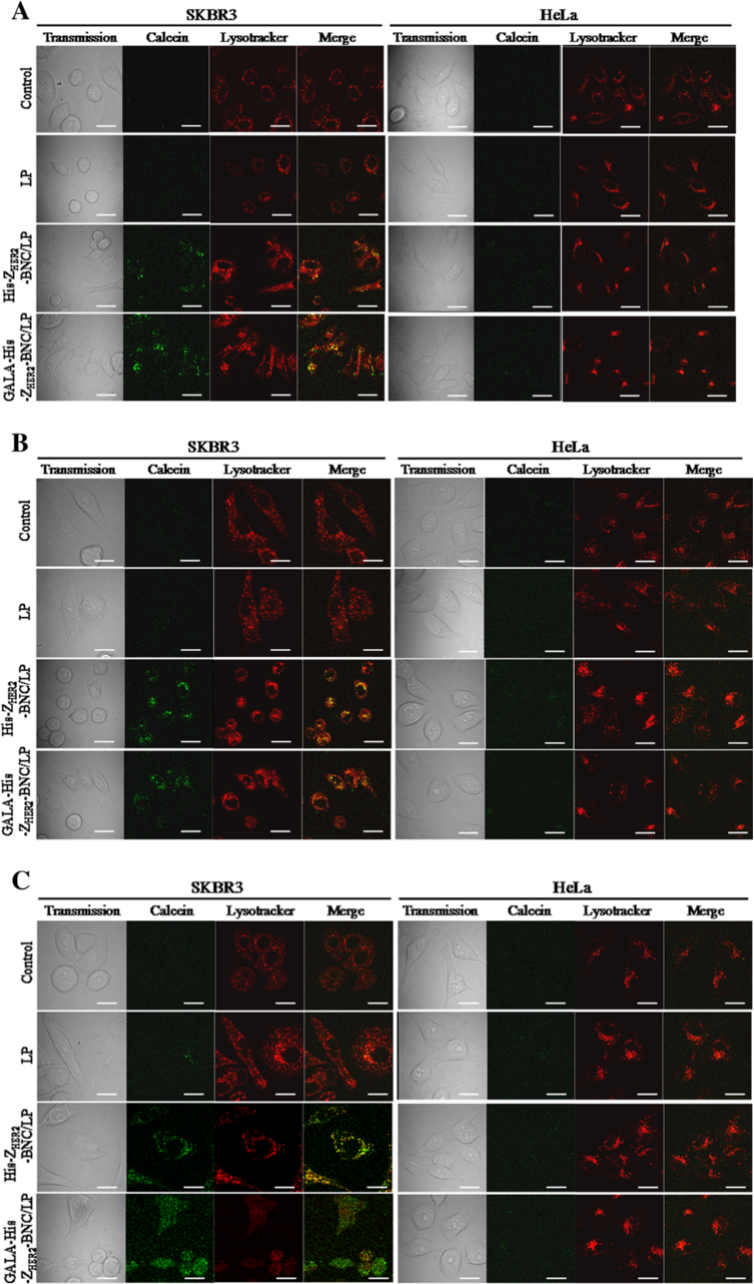
**Fluorescence images of HER2-expressing SKBR3 and HER2-non-expressing HeLa cells treated with LP, His6-Z**_**HER2**_**-BNC/LP, and GALA-His6-Z**_**HER2**_**-BNC/LP encapsulating calcein after incubation for 6 h (A), 24 h (B), and 48 h (C).** SKBR3 cells were maintained in RPMI 1640 medium supplemented with 10% (v/v) FBS at 37°C in 5% CO_2_. HeLa cells were maintained in DMEM medium supplemented with 10% FBS at 37°C in 5% CO_2_. Approximately 5 × 10^4^ SKBR3 or HeLa cells were seeded in 35 mm glass-bottom dishes. Complex carriers of His-Z_HER2_-BNC and GALA-His-Z_HER2_-BNC with LP, in which calcein was incorporated, were prepared. Freeze-dried LP (COATSOME EL-01-A) was dissolved in distilled water (2 ml) containing 100 mM of calcein. After incubation for 1 h at room temperature, gel-filtration chromatography was performed using a PD-10 (GE healthcare). The obtained LP incorporating calcein (100 μl) was added to freeze-dried His-Z_HER2_-BNC and GALA-His-Z_HER2_-BNC (100 μg as protein) and incubated at room temperature for 1 h. After washing with serum-free medium, 7.5 μl of the carriers containing calcein were added to 200 μl of the medium and then the cells were cultured for 1 h. After washing with serum-free medium twice, cells were incubated with FBS-containing medium for 5 h, 23 h and 47 h. Cells were observed using a LSM 5 PASCAL laser scanning confocal microscope (Carl Zeiss, Oberkochen, Germany) with a 63-fold oil immersion objective lens with excitation using the 488-nm line of an argon laser and emission collection using a 505–530 nm band pass filter for calcein and the 543-nm line of an He-Ne laser and emission collection using a 560-nm long-pass filter for the Lysotracker Scale bar, 20 μm.

Next, to determine if the GALA-His-Z_HER2_-BNC had the ability of endosomal escape, we prepared a complex conjugating a GALA-His-Z_HER2_-BNC with anionic LP (COATSOME EL-01-A) that has never shown the ability of endosomal escape (GALA-His-Z_HER2_-BNC/LP). The complex carriers were prepared by referring to the previously described BNC/LP conjugation method with some modifications [17]. To visualize the destination of the particle inclusions, a green fluorescent compound (calcein) was encapsulated into the LP as an inclusion. Then, three types of particles incorporating calcein (LP, His-Z_HER2_-BNC/LP and GALA-His-Z_HER2_-BNC/LP) were added to HER2-positive SKBR3 cells (human breast carcinoma) [18] and HER2-negative HeLa cells (human cervical carcinoma) [19]. The cellular kinetics was observed using a confocal laser scanning microscope (CLSM) after staining endosomes with red fluorescent Lysotracker ® Red DND-99 (Invitrogen Life Technologies, Carlsbad, CA, USA) (Figure 3). When the calcein and Lysotracker fluorescence merged, yellow regions indicated the endosome localization of particles containing calcein.

After incubation for 6 h (Figure 3A), the cellular uptake of His-Z_HER2_-BNC/LP and GALA-His-Z_HER2_-BNC/LP was observed in HER2-expressing SKBR3 cells. Both merged images showed yellow fluorescence, indicating that calcein were localized in endosomes with no occurrence of inclusion in the endosomal escape. Similar trends were observed after incubation for 24 h (Figure 3B). After incubation for 48 h (Figure 3C), the His-Z_HER2_-BNC/LP remained trapped in the endosome. However, the GALA-His-Z_HER2_-BNC/LP showed clear green fluorescence in the cytoplasm, indicating that calcein was successfully released from the endosomes to the cytoplasm. Red fluorescence derived from the Lysotracker was rarely observed in the case with the GALA-His-Z_HER2_-BNC/LP, suggesting that the GALA peptide had destroyed the endosomal membranes through the process of endosomal escape, and, therefore, that the endosomes had diminished. It has been reported that endosomal escape of GALA-displaying LP was observed after incubation for 18 h [20,21]. Because the uptake mechanisms were different between the previous report (Transferrin receptor-mediated endocytosis) and the current study (HER2 receptor-mediated endocytosis), it seemed that the GALA-His-Z_HER2_-BNC/LP required the longer time for releasing the calsein into the cytoplasm. In addition, the difference in the contact time to cells (previous studies, 3 h [20] and 18 h [21]; and current study, 1 h) might be also affected to the endosomal escape. We additionally showed that the GALA-His-Z_HER2_-BNC/LP was never introduced into the HER2-non-expressing HeLa cells (Figure 3A–C). Thus, we successfully demonstrated that the genetic fusion of a GALA peptide into a Z_HER2_-BNC enabled endosomal escape without the inclusion of a specialized LP such as DOPE.

**Figure 4:**
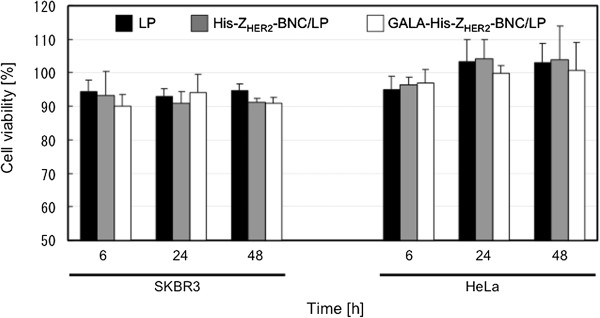
**Cell viability of SKBR3 and HeLa treated with LP (black bars), His-Z**_**HER2**_**-BNC/LP (gray bars) and GALA-His-Z**_**HER2**_**-BNC/LP (white bars).** Approximately 5 × 10^3^ SKBR3 or HeLa cells were seeded in 96-well plate. LP, His-Z_HER2_-BNC/LP and GALA-His-Z_HER2_-BNC/LP containing calcein (3.75 μl) were added to 100 μl of the medium and then the cells were cultured for 1 h. After washing with serum-free medium twice, cells were incubated with FBS-containing medium for 5 h, 23 h and 47 h. Cell viability was measured using Cell Counting Kit-8 (DOJINDO LABORATORIES, Kumamoto, Japan) according to the manufacturer’s instructions.

To evaluate the toxicity of particles, cell viability treated with each particle was measured (Figure 4). It was confirmed that the cell viability of SKBR3 treated with each particle was over 90%. Because the cell viability treated with positively-charged LP displaying GALA was reported as less than 60% [5], the toxicity of each particle (negatively-charged LP, His-Z_HER2_-BNC/LP and GALA-His-Z_HER2_-BNC/LP) was almost similar and seemed to be low. Thus, it has demonstrated that the display of GALA peptide on the BNC never showed the negative effect on the cell viability.

In the present study, a Z_HER2_-BNC acquired the ability of endosomal escape by displaying GALA on the surface of the particle, which enhanced its availability as a drug delivery carrier to the target cytoplasm. Furthermore, the genetic fusion of a GALA peptide makes the ability of endosomal escape possible for chemically or microbially (*E. coli*) synthesized GALA peptides. These findings are valuable as a demonstration of the ability of endosomal escape using various virus-like particles that could help facilitate the functional expression of inclusions.

## Abbreviations

HER2: Human epidermal growth factor receptor 2; BNC: Bio-nanocapsule; HBV: Hepatitis B virus; DOPE: 1,2-dioleoyl-sn-glycero-3-phosphoethanolamine; DDS: Drug delivery system; HA2: Hemagglutinin 2; HBsAg: Hepatitis B virus surface antigen; LP: Liposome; DLS: Dynamic light scattering; CLSM: Confocal laser scanning microscope; PAGE: Poly-acrylamide gel electrophoresis; DMEM: Dulbecco’s modified eagle medium; FBS: Fetal bovine serum.

## Competing interests

The authors declare that they have no competing interests.

## Authors’ contributions

Conceived and designed the experiments: YN, KT, JI and CO. Performed the experiments: YN, KT and RE. Analyzed the data: YN and KT. Wrote the paper: YN and JI. Supervised the whole work: AK. All authors have read and approved the final manuscript.
